# Microhardness and shear bond-strength of carious dentin after fluorescence-aided or conventionally excavation: (An *in-vitro* comparison)

**DOI:** 10.4317/jced.53592

**Published:** 2018-07-01

**Authors:** Uzay Koç-Vural, Esra Ergin, Sevil Gurgan

**Affiliations:** 1 Hacettepe University, Department of Restorative Dentistry, 06100 Sıhhıye/Ankara

## Abstract

**Background:**

To compare the fluorescence-aided and conventionally excavated dentin with microhardness and shear bond strength(SBS) tests.

**Material and Methods:**

Twenty-four teeth with dentin caries were bisected through the center of the lesion into two halves. Forty-eight dentin specimens were embedded and mounted in an acrylic resin. All carious tissue was removed and classified as caries free using conventional visual tactile criteria. Then half of the specimens(n=24) were reinspected with fluorescence-aided caries excavation light(FACE) (FaceLight, W&H Dentalwerk, Bürmoos GmbH, Austria). Specimens were subjected to microhardness and shear bond strength testings. The fracture mode analysis was also performed. The data were compared with Student’s t test and Chi-square test.

**Results:**

Residual caries was observed in 2 out of 24 conventionally excavated specimens with FACE inspection(*p*>0.05). Mean Vickers hardness of the dentin was 61.5±5 in the FACE group and 70.3±3 kg/mm2 in the conventionally excavated group(*p*>0.05). The mean SBS value of FACE group was 11.42±1.63 MPa and 18.27±1.43 MPa in conventionally excavated group. There was no statistically significant difference between conventional and FACE groups for microhardness and SBS tests(*p*>0.05). There were also no significant differences on the fracture mode distributions of the groups(*p*>0.05).

**Conclusions:**

FACE method could be considered as a promising technique for removing infected dentin.

** Key words:**FACE, conventional excavation, residual caries detection, shear bond strength, microhardness.

## Introduction

Caries excavation has been still regarded as a confusing procedure for the dental practitioners. The concept of minimally invasive dentistry requires the selective removal of carious dentin which consists two layers (1). The outer infected layer, which is heavily infected by bacteria and irreversibly denatured has to be removed while the only partially demineralized inner affected layer, which has the potential of remineralization, should be preserved during caries excavation process (2).

Many excavation methods have currently been used for the removal of infected dentin. In the conventional excavation technique, dentists determine the endpoint of the excavation by the hardness and color of the dentin (3). Clinicians keep excavating carious dentin until reaching to the mineralized layer, considering the depth of excavation based on the dentin hardness and color. However, up to date, no certain criteria have been specified yet to discriminate the carious dentin layers. In the traditional visual-tactile inspection technique performed with a mirror and an explorer; it is considered that “the probe should not stick in the dentin and should not give a tug-back sensation” (4). However, the demineralized affected dentin, which can remineralize in time, could be sacrificed during the conventional excavation (5-7). Since this sensation is highly subjective, dye staining has been developed to make this procedure more objective by indicating only demineralized tissues (8,9). It has been previously reported that dye staining and bacterial penetration are different phenomena since dye staining might be a poor indicator for the discrimination of carious dentine layers (10). Therefore, alternative dentin caries removal methods using optical aids have been recently developed (11).

Fluorescence aided caries excavation (FACE), a novel caries excavation system has been claimed to be an objective method in the removal of infected dentin (12). In the FACE system, sound dentin fluorescences green after illuminated with violet light, whereas bacterially infected dentin emits red-orange fluorescence. When an exposed cavity is illuminated by the violet light, which causes the dental hard tissue to autofluorescence (wavelength of 405 nm), the porphyrins exuded by oral bacteria show a red fluorescence, indicating the essential areas for caries excavation (12,13). Dental hard tissues fluorescence green and carious dental hard tissues fluorescence orange-red under the FACE light. FACE has been shown to be more effective than conventional excavation in the removal of infected dentin *in vitro* (14-16).

Until now, little attention has been paid to the relation between hardness and the bond strength to dentin inspected with FACE. Thus, the aim of this study was to evaluate the microhardness and shear bond strength (SBS) of dentin, after inspected by FACE and to compare this technique with the visual-tactile method.

## Material and Methods

**Figure 1:**
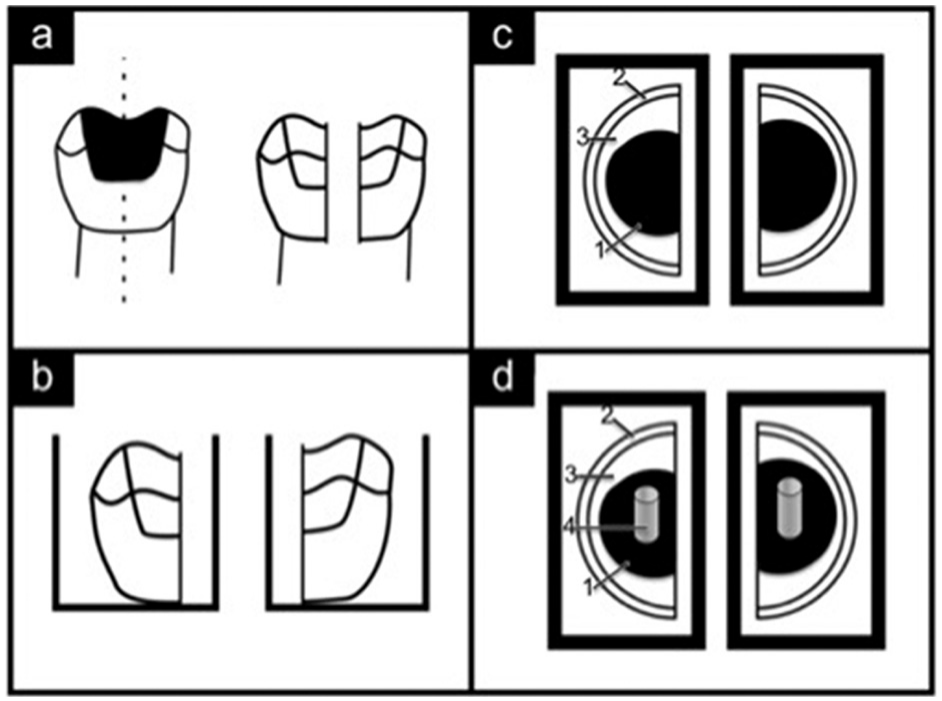
Preparation of the specimens. 1-Caries excavated dentin, 2-Enamel, 3-Sound dentin, 4-Composite resin disc. (a) Teeth were bisected through the center of the lesion in the inciso-apical direction into the two halves. (b) The obtained sections were embedded in a chemically cured acrylic resin. (c) The occlusal surfaces were exposed (d) A resin composite disc was placed over dentin for SBS testing.

The experimental procedures were approved by the local ethics committee (GO 14/106). Freshly extracted, permanent maxillary or mandibular molars with occlusal caries lesions on the central fissure were collected and stored in 0.05 % formaldehyde solution at 4°C up to 1 month. Among those, the teeth having; a) caries lesions limited on enamel or reaching up to the pulp chamber, b) any restoration, c) sign of tetracycline staining and d) any cracks were excluded. Finally 24 teeth with visually similar sized caries lesions were selected and bisected through the center of the lesion in the occluso-apical direction to minimize the anatomical variations (Fig. 1a). The obtained sections were embedded in a chemically cured acrylic resin (Fig. 1b) (Vertex, Vertex Dental, Zeist The Netherlands), so that the occlusal surfaces could be exposed (Fig. 1c).

Then the specimens were divided into 2 groups representing similar lesion morphology (n=24). This allowed the allocation of the teeth into two groups of 24 teeth each, so that each group had the similar lesions before the excavation (Fig. 1c). The specimens were soaked in distilled water immediately at the dough stage of resin to avoid the harmful effect of temperature rise during polymerization, and were kept in distilled water at room temperature until hardness measurements were done within 24 h.

Caries Excavation: One operator performed the caries removal procedures (UKV) to minimize variations. Carious dentine of all specimens (n=48) was excavated conventionally. Soft dentin was removed by the help of round steel burs with different sizes without water-cooling in a low speed hand-piece and straight spoon excavators. The carious dentine was removed until the operator assessed the specimen as “free from caries” using the visual-tactile criteria. The preparation was finalized when the probe did not give a tug-back sensation, and clinically seemed sound. The hard dentin was preserved.

Half of the conventionally excavated specimens, having similar lesions with the remaining 24 specimens, were inspected with FACE light (FaceLight, W&H Dentalwerk, Bürmoos GmbH, Austria) technique. This device generated fluorescence violet light (405 nm) by the help of 100-130-watt xenon discharge lamp to inspect the surfaces of the specimens (n=24). The room was darkened during the inspection procedure. Red-orange colored areas were further excavated until the transform was completed from orange-red to the green fluorescencing area.

**Figure 2:**
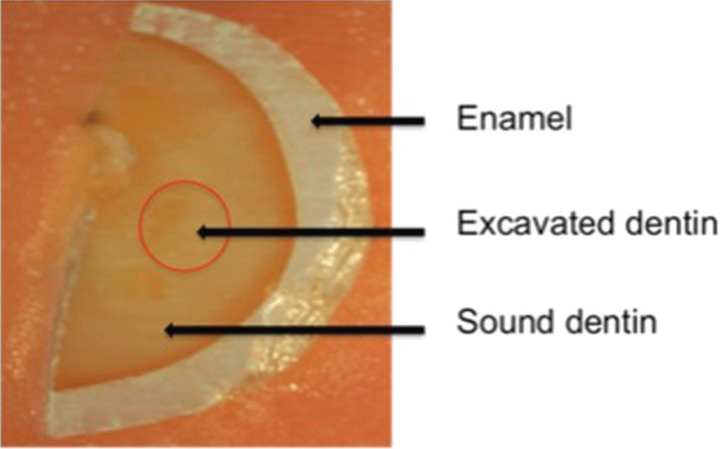
A prepared specimen.

Hardness test: All hardness measurements were performed on the excavated surfaces of the specimens. An adhesive tape was adapted to the deepest cut surface of each specimen to ensure the excavated area and the surface was flattened by hand-polishing with 800,1200, 2500, and 4000 grid wet silicon carbide abrasive papers, respectively (Fig. 2). After the adhesive tape was removed, they were dried briefly using compressed air before the hardness test.

Vickers hardness tester (HMV, Shimadzu Corporation, Japan) was used to obtain precise hardness profiles of the excavated dentin surfaces. A load of 1 Newton (100 g) was applied for 15 s to produce the indentations. The Vickers hardness numbers (VHNs) were measured at five points in dentin with a minimum distance of 40 μm. Each line of indentations was placed in the area to ensure the presence of the excavated dentin.

Shear-bond strength (SBS) test: Following the hardness test, the excavated dentin surfaces were etched for 15 seconds with 37%phosphoric acid gel (Condac 37, FGM, Setubal, Portugal). After rinsing for 10 seconds, the etched dentin surfaces were gently dried with an absorbent paper to produce a visibly moist and not desiccated surface. Gluma 2 bond (Heraeus Kulzer, Hanau, Germany) was applied with the applicator tips over the dry dentin surface according to manufacturer’s instructions and light cured for 20 seconds. Resin composite (Charisma diamond, Heraeus-Kulzer, Hanau, Germany) discs were built (Fig. 1d) as two increments by light curing each increment for 20 seconds,with the help of a teflon jig (Ultradent Products Inc., South Jordan, UT) which was adapted perpendicular to the excavated surfaces. The dimensions of the resin composite discs were 2.38 mm in diameter and 2.5 mm in length. The specimens were then transferred to an Instron testing machine (Lloyd, Model LRX, England) and subjected to SBS test. The specimens were positioned in the device so that the shearing stamp would load the composite cylinder at a 90° angle with a crosshead speed of 1mm/s and a cell load capacity of 1 kN until failure.

Fracture analysis was carried out at X40 magnification under stereomicroscope (Olympus SZ61, Tokyo, Japan). Fractures either in the dental tissue or in the composite was classified as cohesive failure. If dentin tubules were exposed and the residues of either the adhesive and/or the composite were detectable, the specimen was assigned to the group of mixed fractures/failure. Adhesive fracture between adhesive agent and dental hard tissue was classified as adhesive failure.

Statistical analysis: All statistical analyses were carried out using SPSS, version 21.0 (IBM SPSS, Chicago, IL, USA), and the significance level was set to 5% for all tests. Shapiro-Wilk test showed that the microhardness values of the dentin were normally distributed. The comparisons of microhardness and SBS between the FACE and conventionally excavated groups were done using “T test” The frequencies were also calculated for qualitative variables.

## Results

**Figure 3:**
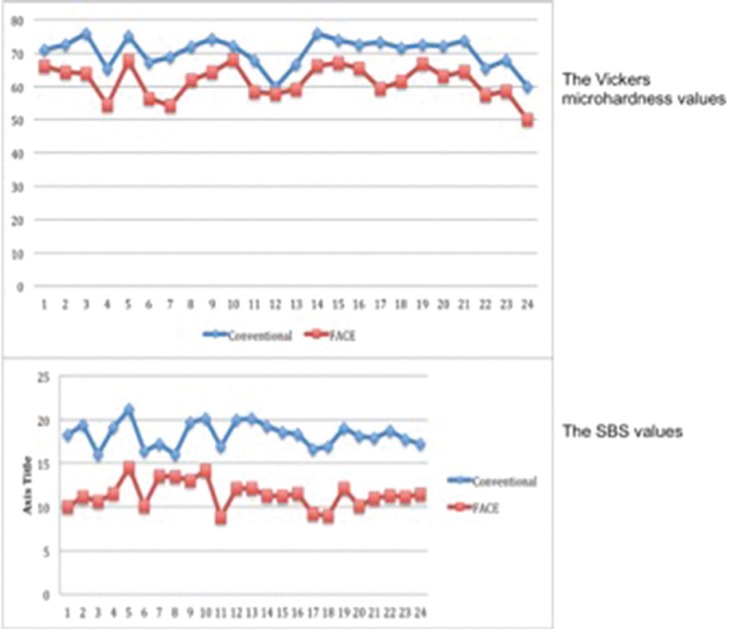
The Vickers microhardness and SBS values.

The mean Vickers microhardness and standard deviations of dentin following conventional excavation and FACE were 70.3±3 and 61.5±5, respectively (Fig. 3,  Table 1). No statistically significant difference was found between conventional and FACE methods in the microhardness testing (*p*>0.05). The mean SBS of conventional excavation was 18.27 ± 1.43 MPa whereas FACE group exhibited a mean SBS of 11.42 ± 1.63 MPa (Fig. 3,  Table 1). Although the conventional excavation yielded to present higher SBS value, the difference between two groups was not statistically significant (*p*>0.05). The results are summarized in  Table 1.

**Table 1:**
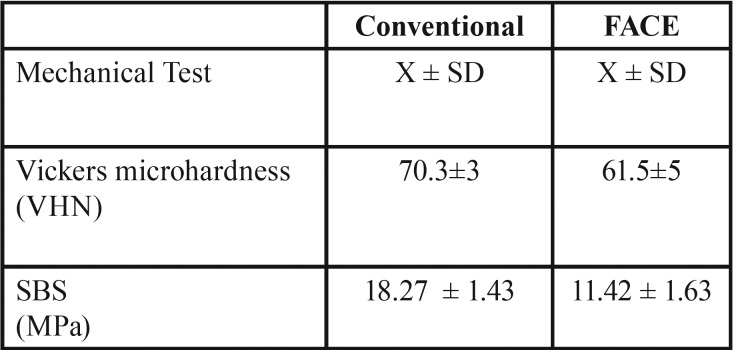
Mean values of mechanic tests.

The majority of all specimens showed adhesive failure. Only 2 specimens from conventional group showed cohesive failure, which was not significant (*p*>0.05). The results are presented in  Table 2.

**Table 2:**
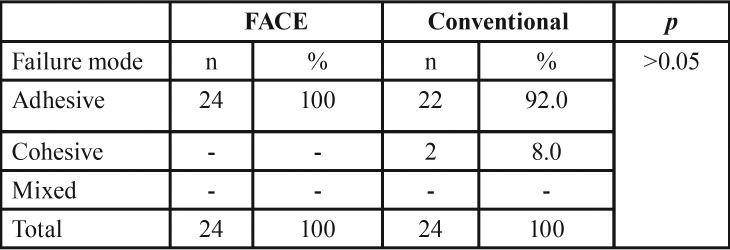
Results of the fracture mode analyses.

## Discussion

This study introduced a novel caries-detection method that is potentially capable of objectively differentiating the caries-infected, affected and sound dentin. This method may lead to more precise and minimal sound dentin removal after excavation. Moreover, present study evaluated the excavated dentin areas by the help of dentin microhardness and SBS.

The knowledge of the mechanical properties of human dentin can be helpful to assess the benefits of different treatment alternatives. Microhardness tests, based on the induced permanent surface deformation that remains after removal of the load (17,18), has been used in *in vitro* studies to differentiate the sound and carious dentin or the effects of various types of treatments on dental hard tissues. It offers comparable and objective relative dentine hardness values, which gives direct clinical translational value to operators (19-21). The most commonly used approaches were the Knoop and Vickers hardness tests. Although the optical evaluation of Knoop and Vickers indenter impressions are subjective when transparent materials used such as tooth hard tissues, at lower loads, tha data variation errors increase at small measurement and viscoelastic materials exhibit a time dependent elastic recovery leading to additional variations (22). To eliminate these disadvantages, nanoindenters was used to evaluate the nanomechanical properties of dental hard tissues however its high cost limits the its availability (23).

It was previously reported that the mean Vickers microhardness of dentin following conventional rotary instruments were ranging from 50 to 70 VHN and sound dentin provides 66.94VHN (24-26). In this study, the mean Vickers microhardness of dentin following conventional rotary instrument was found 70.3±3. Similar to the study of Lai *et al.* (12), present study found that FACE method resulted in significantly lower microhardness than those obtained by conventionally excavated.

In this study, although no significant differences were observed between the microhardness value of two detecting methods, FACE group presented slightly lower microhardness. This decrease may be explained by the selective removal of caries-infected dentin, leaving more caries-affected dentin tissue, has a lower hardness value. The hardness values at these locations close to the pulp was also reported to be lower compared to superficial dentin, mid-dentin (12,27,28). The selective removal of visualized porphyrins may also lead to the preparation of deeper areas in dentin according to the density and orientation of the dentin tubules where the dentin hardness varies because of different mineralizations. However, variations in the dentin composition and the density of dentin tubules makes bonding to dentin a challenging procedure.

Kumari *et al.* (29) investigated the bond strength of nano-composite resin to superficial/deep dentin and reported a significant decrease in bond strength values from superficial to deep dentin.

In this study, FACE group showed lower SBS results than conventional group, which correlates with the current literature. We also have to take into consideration that, locally more tissue removal via FACE as only bacteria products-contaminated dentin was illuminated and removed and concluded by microhardness test, in this study. The composition of dentin substrate consists of 50% minerals, 20% water, and 30% organic matrix (29). However, it is well known that, as the dentin deepens, this composition changes, number of tubules and amount of water increases. The adhesive resin used in this study contains ethanol as a solvent and after the evaporation of the solvent no movement of liquid must be detected. In the presence of undesirable water arising in dentin tubules may impair adhesion between composite resin and dentin. Therefore, lower bond strength was achieved in comparison with the superficial dentin. However, although, no difference was observed between the groups in this study, almost all the test specimens showed adhesive fracture in correlation with the current literature (29,30).

Despite the paradigm of minimal-invasive dentistry, the degree of carious dentin that has to be removed from a cavity is still a matter of discussion and it is still an unsolved problem for the practitioner. Yet, it is not known as to how far the dentin must be removed. Although, FACE seems to be promising on minimizing the unnecessary risk of over excavation by detecting only infected dentin and preventing affected dentin, this technique needs to be validated by further in-vivo/vitro investigations.
